# CAVIN2/SDPR Functioned as a Tumor Suppressor in Lung Adenocarcinoma from Systematic Analysis of Caveolae-Related Genes and Experimental Validation

**DOI:** 10.7150/jca.84567

**Published:** 2023-07-03

**Authors:** Keyun Zhu, Baichuan Wang, Yingxi Li, Yue Yu, Zhaohui Chen, Haoran Yue, Qingxiang Meng, Dongchen Tian, Xiaofeng Liu, Weiyu Shen, Yao Tian

**Affiliations:** 1 Department of Thoracic Surgery, Ningbo Medical Centre Lihuili Hospital, Ningbo University, Ningbo, Zhejiang, P. R. China, 315040.; 2 Anhui Medical University Clinical College of Chest, Hefei, Anhui Province, P. R. China, 230022.; 3 Anhui Chest Hospital, Hefei, Anhui Province, P. R. China, 230022.; 4 Key Laboratory of Immune Microenvironment and Disease (Ministry of Education), Tianjin Medical University, Tianjin, P. R. China, 300070.; 5 Key Laboratory of Cancer Prevention and Therapy, Tianjin's Clinical Research Center for Cancer, Tianjin Medical University Cancer Institute and Hospital, Tianjin, P. R. China, 300060.; 6 Department of General Surgery, Tianjin Medical University General Hospital, Tianjin, P. R. China, 300052.

**Keywords:** lung cancer, biomarkers, LUAD, SDPR, cell cycle

## Abstract

**Background:** Caveolae-Related Genes include caveolins and cavins, which are the main component of the fossa and, play important roles in a variety of physiological and pathological processes. Although increasing evidence indicated that caveolins (CAVs) and cavins (CAVINs) are involved in carcinogenesis and progression, their clinical significance and biological function in lung cancer are still limited.

**Methods:** We investigated the expression of CAVs and CAVINs at transcriptional levels using Oncomine and Gene Expression Profiling Interactive Analysis. The protein and mRNA expression levels of CAVs and CAVINs were determined by the human protein atlas website and our surgically resected samples, respectively. The clinical value of prognostic prediction based on the expression of CAVs and CAVINs was also assessed. cBioPortal, GeneMANIA and STRING were used to analyze the molecular characteristics of CAVs and CAVINs in lung adenocarcinoma (LUAD) comprehensively. Finally, we investigated the effect of CAVIN2/SDPR (serum deprivation protein response) on LUAD cells with biological experiments in vitro.

**Results:** The expression of CAV1/2 and CAVIN1/2/3 were significantly downregulated in LUAD and lung squamous cell carcinoma (LUSC). The patients with high expression of CAV1, CAV2, CAV3, CAVIN1 and CAVIN2/SDPR were tightly correlated with a better prognosis in LUAD, while no statistical significances in LUSC. Further, our results found that CAVIN2/SDPR can be identified as a prognostic biomarker independent of other CAVINs in patients with LUAD. Mechanically, the overexpression of CAVIN2/SDPR inhibited cell proliferation and migration owing to the cell apoptosis induction and cell cycle arrest at S phase in LUAD cells.

**Conclusions:** CAVIN2/SDPR functioned as a tumor suppressor, and was able to serve as prognostic biomarkers in precision medicine of LUAD. Mechanically, overexpression of CAVIN2/SDPR inhibited cell proliferation by inducing cell apoptosis and S phase arrest in LUAD cells.

## Introduction

Lung cancer is the third most common cancer and the leading cause of cancer-related mortality worldwide 1. Non-small cell lung carcinoma, includes several histological subtypes, such as adenocarcinoma (LUAD), squamous cell carcinoma (LUSC), adenosquamous carcinoma, and large cell carcinoma 2. Among which, LUAD is the most diagnosed histological subtype, accounting for approximately 40% of all lung cancers and still arising 3. Despite many promising new therapies continuously comes up, such as immunotherapy and targeted therapies. The treatment of lung cancer still faces many difficulties due to gene mutation, tumor heterogeneity, and drug resistance 4. Therefore, it is of great clinical value to identify molecular targets related to the tumor initiation, progression, metastasis and prognosis of lung cancer.

Caveolae are flask-shaped invaginations of cell membrane, which play pivotal roles in a variety of cellular response processes, including signal transduction 5, nutrients transportation 6, pathogens endocytosis 7, cell cycle regulation 8 and so on. The formation of Caveolae depends on integral membrane proteins termed caveolins and cavins. There are seven family members of caveolins and cavins: caveolin-1 (CAV1), caveolin-2 (CAV2), caveolin-3 (CAV3), cavin-1 (CAVIN1), cavin-2 (CAVIN2, also known as serum deprivation protein response, SDPR), cavin-3 (CAVIN3) and cavin-4 (CAVIN4) 9. It has been reported that caveolae proteins (caveolins and cavins) are involved in the tumorigenesis and tumor progression, such as breast cancer 10, 11, colon cancer 12, 13 and prostate cancer 14. However, the roles of caveolae proteins in lung cancer are largely unknown.

In the family members of caveolins, CAV1, a main component of caveolae, exhibited anti-tumor functions in lung cancers, which was associated with cell proliferation, migration, apoptosis and drug resistance of lung cancer 15. While CAV2 was reported to can directly interact with CAV1 16. CAV3 is predominantly expressed in muscle tissues and few studies have reported its function in cancer 17, 18. According to cavin family members, they are re-sponsible for the caveolae formation. Researchers found that CAVIN1 was proved as a biomarker of by label-free proteomics and related to the EGFR pathway 19. CAVIN2/SDPR is mainly responsible for controlling the change of the caveolae shape. Furthermore, CAVIN2/SDPR has also been reported as a tumor suppressor in a variety of cancers 20, 21. Our group previously found that CAVIN2/SDPR depletion can induce epithelial-mesenchymal transition in breast cancer cells by activating TGF-β signaling pathway 22. In contrast to CAVIN1 and CAVIN2/SDPR, the role of CAVIN3 in caveolae formation hasn't been well established. The loss of CAVIN3 in lung cancer appears to be the consequence of DNA methylation and subsequent gene silencing, indicating that CAVIN3 functions as a tumor suppressor candidate 23. Given that cavins proteins are required in conjunction with caveolins for caveolae formation, their roles in tumor regulation will still require more exploration.

In order to investigate the relationship between caveolae proteins and clinical characteristics of patients in lung cancer, we firstly performed integrated bioinformatic analysis using online database. Secondly, we determined the expression of CAVs and CAVINs at protein and mRNA levels and evaluated the clinical prognostic value in patients of lung cancer. We further conducted the genetic alteration, interaction network, and functional enrichment of CAVs and CAVINs in LUAD. Finally, experiments in vitro confirmed that CAVIN2/SDPR, functioned as a tumor suppressor, inhibited cell proliferation and migration in LUAD cells by inducing cell apoptosis and cell cycle arrest at S phase. In summary, our findings provide sufficient evidence for the precise therapy towards lung cancer by targeting CAVs and CAVINs.

## Materials and Methods

### Oncomine

The transcriptional levels of CAVs and CAVINs in diverse cancer types were compared through analysis in Oncomine (https://www.oncomine.org), a public online cancer database for genome-wide expression analysis 24. Student's t test was used to compare the mRNA levels of CAV and CAVIN in cancer samples with those in normal samples. Statistical significance values and fold change were defined as P-value <0.01 and 2, respectively, and the top 10% genes were set as the significance thresholds.

### Gene Expression Profiling Interactive Analysis (GEPIA)

GEPIA (http://gepia.cancer-pku.cn) is a tool for analyzing RNA-Seq data based on The Cancer Genome Atlas (TCGA) and GTEx 25, which can be used for multidimensional analysis. We performed a differential mRNA expression analysis of tumor and normal tissues, pathological stage analysis of CAVs and CAVINs in the LUAD and LUSC patients. The individualized studies were conducted under standard processing requirements.

### UALCAN

UALCAN (http://ualcan.path.uab.edu/analysis.html), a comprehensive web resource, provides analyses based on TCGA data 26. In this study, we further analyzed the relationship between the expression levels of different CAVs and CAVINs and the individual cancer stages via UALCAN database.

### Human Protein Atlas

Human Protein Atlas (https://www.proteinatlas.org) includes immunohistochemistry expression data 27. The protein expression of CAVs and CAVINs in LUAD and LUSC and tumor tissues were performed using immunohistochemical image analysis. To explore the expression of CAVs and CAVINs, the inclusion criteria is the patients suffering from LUAD, LUSC and normal samples. Simultaneously, one sample which is neoplasm malignant in lung with unknown reason is excluded in our study. The extent of staining was scored as the following criteria: (a) percentage of stained cells: 0 (0%), 1 (1%-25%), 2 (26%-75%), 3 (51%-75%), and 4 (> 75%); and (b) staining intensity: 0 (negative staining), 1 (low staining), 2 (medium staining), and 3 (high staining). The final scores were calculated by multiplying the scores of intensities with that of extent.

### RNA extraction, cDNA synthesis and RT-qPCR

According to the manufacturer's guidelines, total RNA was extracted from frozen lung tissues resected surgically using TRIzol reagent (Ambion, USA). cDNA was synthesized by reverse transcription of RNA using PrimeScript RT Master Mix (Takara, Japan). RT-qPCR was carried out using pre-designed primers according to the Manufacturer's instructions (Takara, Japan) with a Bio-Rad CFX96 system. The primers sequences were shown in Table S1.

### Immunohistochemistry

The tumor samples and the paired normal samples in our hospital were patients suffering from LUAD or LUSC. Tissue sections were deparaffinized, rehydrated, and permeated using Triton X 100 (T8200, Solarbio, Beijing, China) and followed by antigen retrieval using EDTA Antigen Retrieval solution (c1034, Solarbio, Beijing, China). The sections were incubated with Anti-CAV1 antibody (ab32577, Abcam, UK), Anti-CAV2 antibody (ab79397, Abcam, UK), Anti-CAVIN1 antibody (ab48824, Abcam, UK), Anti-CAVIN2 antibody (ab76867, Abcam, UK), Anti-CAVIN3 antibody (ab179923, Abcam, UK) at 4 °C overnight followed by a biotinylated secondary antibody (diluted at 1:200) at RT for 60 min. Then, the sections were stained with DAB staining solution (AR1022, BOSTER Biological Technology, Wuhan, China). To quantitatively evaluate the expression levels of each protein in the samples from patients with LUAD and LUSC, we calculated the percentage of cells stained. The extent of staining was scored as the following criteria: (a) percentage of stained cells: 0 (0%), 1 (1%-25%), 2 (26%-75%), 3 (51%-75%), and 4 (> 75%); and (b) staining intensity: 0 (negative staining), 1 (low staining), 2 (medium staining), and 3 (high staining). The final scores were calculated by multiplying the scores of intensities with that of extent.

### Kaplan-Meier Plotter

The Kaplan-Meier plotter (http://kmplot.com/analysis/) was used to investigate the prognosis of mRNA expression according to gene expression data and survival information of patients with LUAD and LUSC. In this study, samples were divided into high expression group and low expression group according to the median expression level of CAVs and CAVINs.

### cBioPortal

cBioPortal (www.cbioportal.org) is a comprehensive web resource for interactive exploration of multiple cancer genomic datasets 28. Based on the TCGA database, genomic profiles of CAVs and CAVINs were investigated, including mutations, copy number alterations (CNAs) and mRNA expression z-scores (RNA Seq V2 RSEM, ±2).

### GeneMANIA

GeneMANIA (http://www.genemania.org) is a resource rich website containing gene information, analytical gene lists and ranking gene functional analyses with high-precision prediction algorithms 29. We used GeneMANIA to identify the gene interaction network and genetic interactions, pathways, co-expression, co-localization, and protein domain similarity of CAVs and CAVINs.

### STRING

STRING (https://string-db.org/) is a database that searches for interactions between proteins, which includes both physical interactions and functional correlations between proteins 30. A PPI network analysis was conducted to collect and integrate the different expression of CAVs and CAVINs.

### GO and KEGG Analysis

DAVID 6.8 (https://david.ncifcrf.gov/home.jsp) is a comprehensive, functional annotation website for better elucidating the biological function of the submitted genes 31. In our study, the Gene Ontology (GO) enrichment analysis and Kyoto Encyclopedia of Genes and Genomes (KEGG) pathway enrichment analysis of CAVs and CAVINs, as well as their closely related neighbor genes were proceeded. Biological processes (BP), cellular com-ponents (CC), and molecular function (MF) were included in the GO enrichment analysis.

### Cell Culture

H1299, A549, H3122, PC-9, H2228, H358, HCC827 and Calu-3 cell lines were obtained from the American Tissue Culture Collection (ATCC, Manassas, VA, USA). The cells were cultured in RPMI-1640 medium (Gibco, USA) containing 10% fetal bovine serum (FBS), penicillin (100 U/mL) and streptomycin (50 g/mL), in a 5% CO_2_ and humidified atmosphere at 37°C.

### Cell Transfection

The SDPR mammalian expression plasmid was purchased from miaolingbio (Wuhan, China). Cells in logarithmic growth stage were collected and seeded into 6-well plates one day before transfection to make the cell density reach 70-80% at transfection. 2 ug plasmid was transfected into cells using Lipofectamine 8000 (Invitrogen) according to the manufacturer's recommendations.

### Western Blot and Antibodies

Cells were collected and washed with cold PBS and lysed on ice for 30 minutes using SDS lysis buffer supplemented with protease inhibitor cocktail (Roche, Switzerland). The collected protein was denatured in a 95°C water bath for 10 minutes. Equal amounts of proteins (30 ug) were separated using SDS‐PAGE. Then, proteins were transferred to PVDF membranes and blocked with 5% bovine serum albumin, followed by incubation with primary and secondary antibodies. The details of the antibodies used in this study are: Anti-SDPR (ab76867, abcam, USA), Anti-Cyclin A (sc-27168, Santa Cruz, USA), Anti-Cyclin B (sc-166210, Santa Cruz, USA), Anti-Cyclin D (sc-8396, Santa Cruz, USA), Anti-Cyclin E (sc-377100, Santa Cruz, USA), Anti-GAPDH (sc-47724, Santa Cruz, USA).

### Cell Proliferation Assay

For MTT assay, the transfected cells (3×10^3^ / 200 µL /well) were seeded in 96-well plates. Cell viability was examined over the next 4 days. After incubation, 20 μL MTT (5 mg/mL in PBS; Sigma) was added to each well and incubated for 3-4 hours, and the formed formazan crystals were dissolved in 150 µL DMSO (Sigma). The absorbance was measured at 570 nm using a micro-plate auto-reader (Bio-Rad). All results are presented as the mean ± SD of triplicate independent experiments.

For colony formation assay, the transfected cells were seeded into 6-well plates at a density of 1000 cells ⁄ well. After approximately 15 days, the colonies had reached an appropriate size and were stained with crystal violet solution. The number of colonies was counted, and the size of the colonies was recorded.

The EdU assay was detected by the BeyoClick™ EdU-594 Cell Proliferation Kit (Beyotime Biotech. Inc.) according to the manufacturer's protocol. Briefly, after transfection for 48 h, cells were incubated with 25 μM EdU for 12 h before fixation, permeabilization, and EdU staining. The percentage of EdU-positive cells was examined by fluorescence microscopy. The data are presented as the means ± SDs of triplicate dishes in the same experiment.

### Transwell Assay

In summary, 1×10^5^ cells were suspended in 200 µL of serum-free RPMI-1640 and added to the upper chamber of each insert, and 600 µL of RPMI-1640 supplemented with 10% FBS was added to the lower chamber of each insert. After incubation for approximately 24 h at 37 ℃ with 5% CO_2_, the cells were fixed with 4% paraformaldehyde and methanol and stained with 1% crystal violet. Random fields were captured by an optical microscope for cell quantification. All the measurements were detected in triplicate.

### Scratch Assay

For the scratch assay, 2×10^6^ cells were plated in 6-well plate. After the cells were confluent and attached, a 10 μL pipette tip was used to generate an even wound in the petri dishes. The width of the wound was recorded at six random locations at the ap-propriate time points (0, 6, 12 and 24 hours). Data are shown as the means and standard deviations (SDs). Images were captured with a microscope at 10× magnification at 0, 12 and 24 hours.

### Flow Cytometric Analysis

For cell cycle distribution, cells were digested, added to 95% ethanol and left at 4℃ overnight. After centrifugation and washing, cells were stained with 500 μL of propidium iodide (PI; BD Biosciences) and incubated in the dark for 15 minutes.

The cell apoptosis assay was carried out according to the instructions of the FITC Annexin V Apoptosis Detection Kit (BD Pharmingen). Cells were digested and re-sus-pended in 1×binding buffer. The solution was stained in 1.5 mL tubes with 5 μL of FITC annexin V and 5 μL of PI for 15 minutes at room temperature in the dark. All samples were analyzed on a FACS Aria flow cytometer (BD) with CellQuest software, and the data were analyzed by FlowJo software.

### Cell Cycle Synchronization

We synchronized the cells to S and M phases and released cells at different time points after synchronization in order to investigate the effect of SDPR on different cell cycle stages. More specifically, the cells are first synchronized to the S phase by a double thymidine (2 mM) block for 18 h, washed twice with PBS and were incubated in warm medium for 4 h. Following the release of the block, the cells are treated with nocodazole (100 ng/mL) for 12 h to arrest them at M phase. After synchronization, cells were washed twice with PBS to release cells from cell cycle blockages and collected at indicated time points.

## Results

### The expression of CAVs and CAVINs in patients with lung cancer

**Figure 1:**
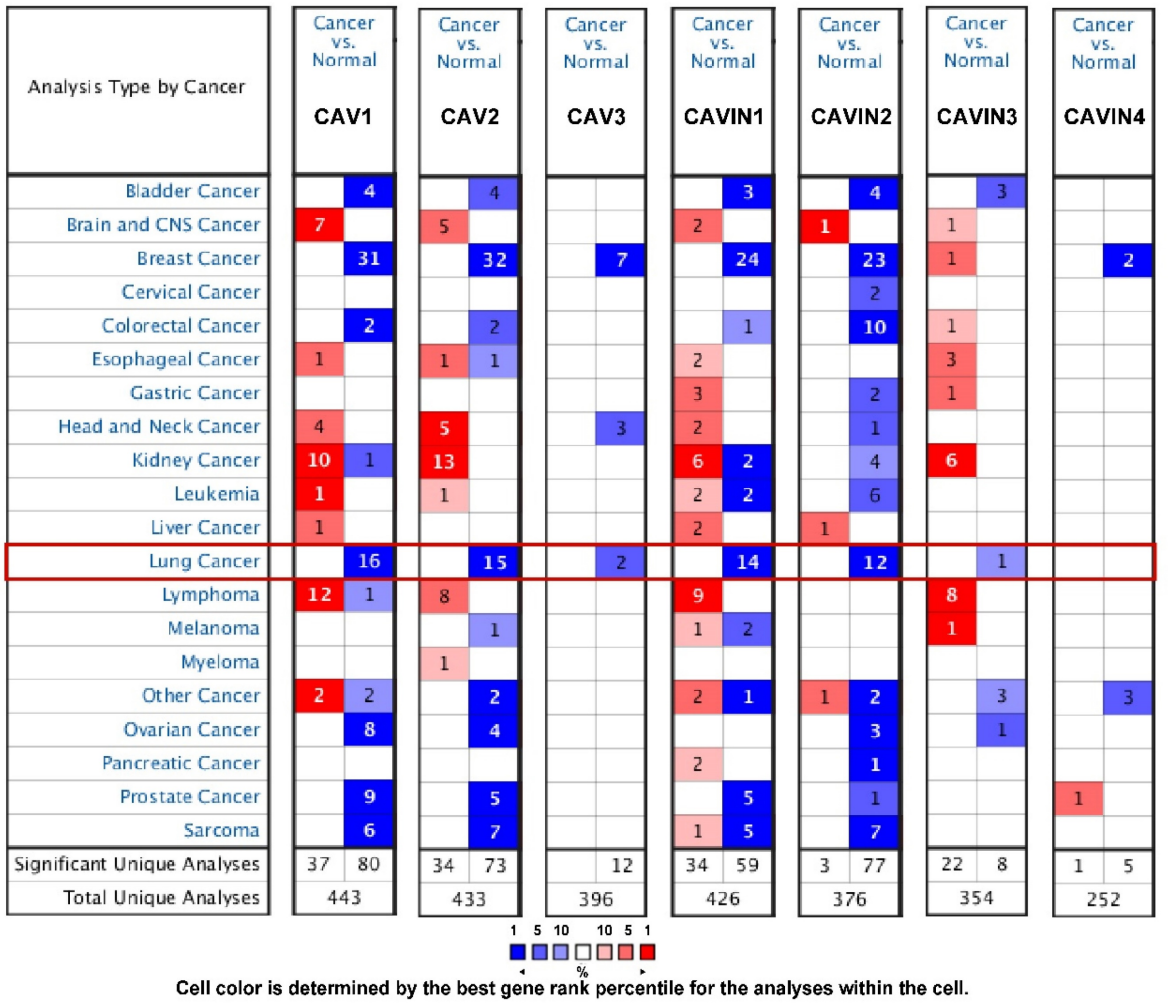
** The transcription levels of CAVs and CAVINs in different types of cancers by Oncomine.** Cancer vs. normal: up-regulated (red) or down-regulated (blue).

The transcriptional levels of CAVs and CAVINs between lung cancer and normal tissues were analyzed using Oncomine database. Figure 1 showed that CAVs and CAVINs were downregulated in lung cancer. In detail, according to the different types of lung cancer, CAV1, CAV2, CAVIN1 and CAVIN2 had a dramatically lower expression in lung adenocarcinoma, squamous cell lung carcinoma and large cell lung carcinoma. Talbot Lung Statistics and Okayama Lung Statistics showed that CAV3 was downregulated in squamous cell lung carcinoma and large cell lung carcinoma. However, CAVIN3 was found to be lower expressed in lung adenocarcinoma in Selamat Lung Statistics (Table S2).

**Figure 2:**
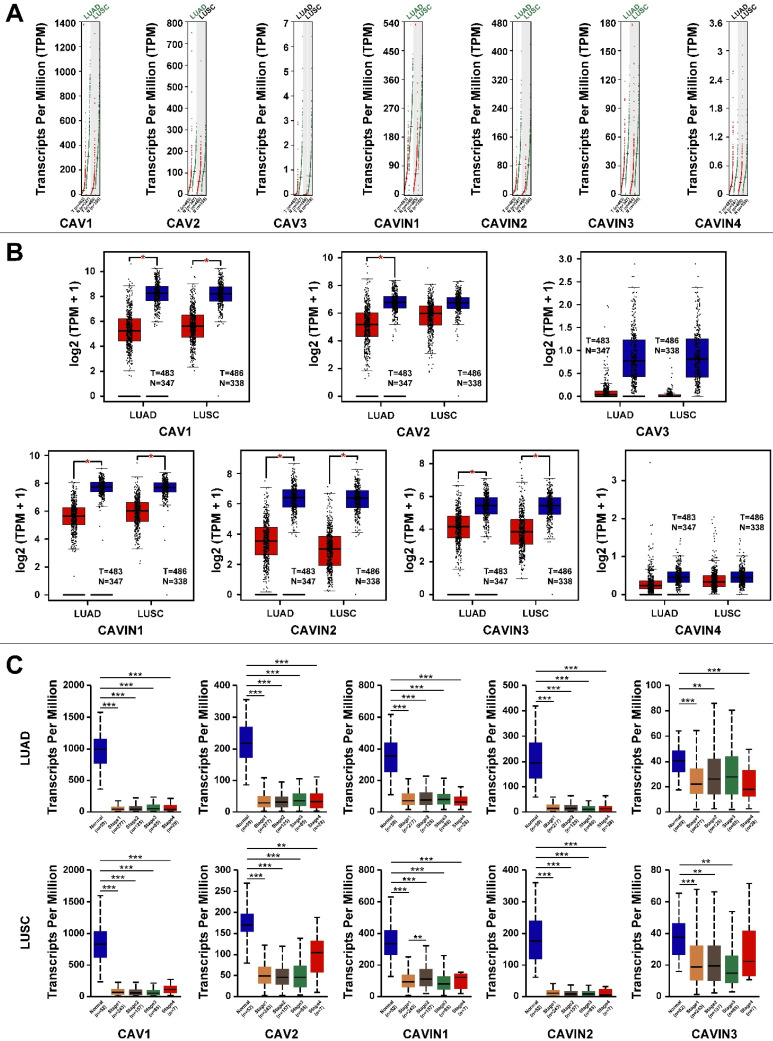
** The expression levels of CAVs and CAVINs in lung cancer.** A and B. The expression of CAVs and CAVINs in lung cancer (T) and normal lung tissues (N) presented by scatter diagram (A) and box plot (B). C. The relationship between CAVs and CAVINs expression levels and clinical stages of patients with lung cancer by GEPIA analysis. *P < 0.05, **P <0.01, ***P < 0.001.

Next, we compared transcriptional levels of CAVs and CAVINs between lung cancer tissues (LUAD and LUSC) and normal tissues using GEPIA. The results exhibited that the expression of CAV1, CAV2, CAVIN1, CAVIN2 and CAVIN3 were significantly lower in LUAD and LUSC than normal, while CAV3 and CAVIN4 had no significant differences (Figure 2A and 2B). Based above, the relationship between the mRNA levels of CAV1/2 and CAVIN1/2/3 and the tumor stage in LUAD and LUSC were performed using UALCAN. However, the expression of CAV1/2 and CAVIN1/2/3 showed no differences in tumor stages both in LUAD and LUSC, which may be due to their extremely low levels in all stages of LUAD and LUSC (Figure 2C).

**Figure 3:**
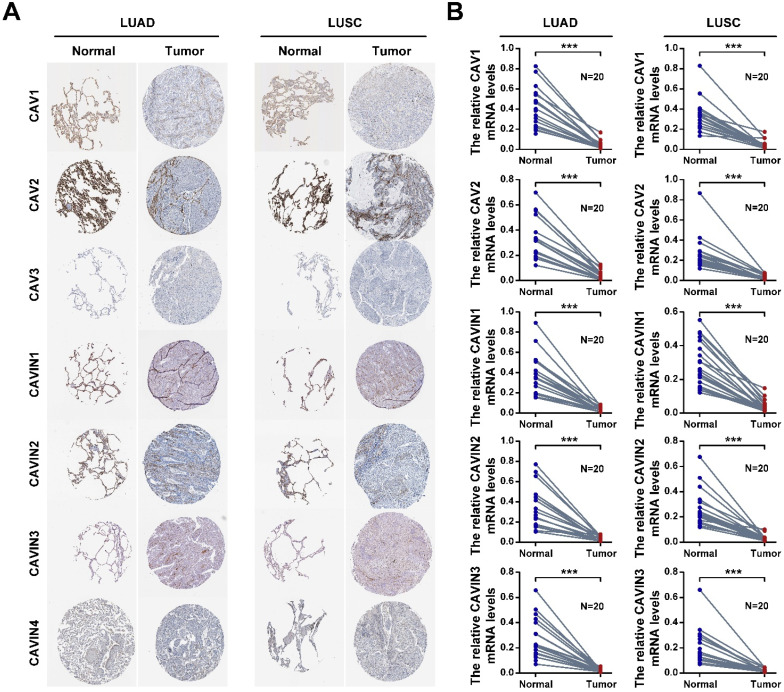
** The expression of CAVs and CAVINs in lung cancer.** A. The protein expression levels of CAVs and CAVINs determined by the human protein atlas. B. The mRNA expression levels of CAVs and CAVINs determined by RT-PCR. **P <0.01, ***P < 0.001.

**Figure 4:**
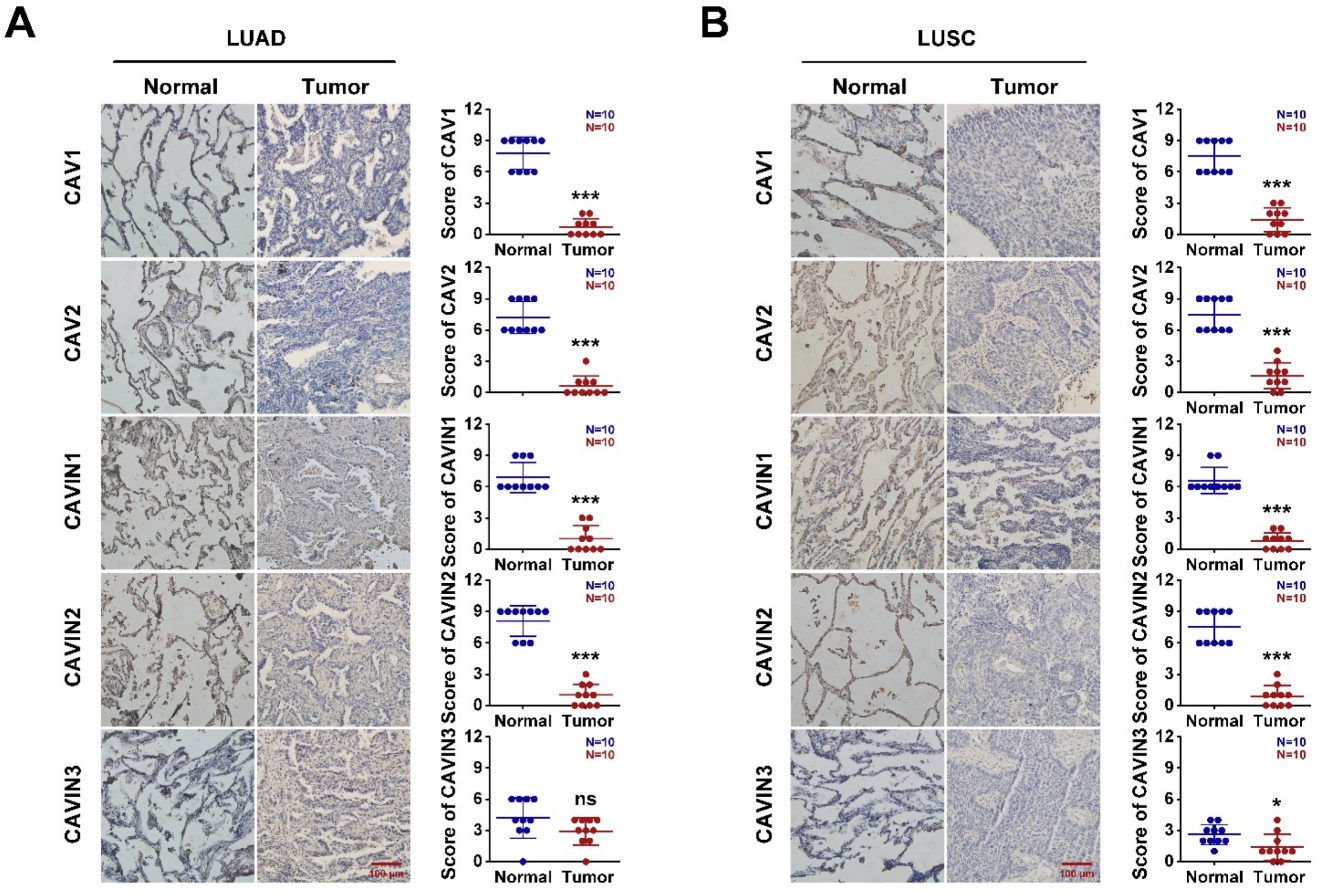
** The expression of CAVs and CAVINs from patients with LUAD (A) and LUSC (B).** *P < 0.05, **P <0.01, ***P < 0.001.

To validate, the expression of CAVs and CAVINs in LUAD and LUSC, the cancer tissues and corresponding normal samples from patients were detected by the immunohistochemistry and RT-PCR. In human protein atlas, CAV1/2 and CAVIN1/2 were significantly downregulated in LUAD and LUSC than normal tissues (Figure 3A and Figure S3). Consistent with the database, the mRNA levels (Figure 3B and Table S3) and protein levels (Figure 4) of CAVs and CAVINs were validated in LUAD and LUSC patients.

### The biological characteristics of CAVs and CAVINs in lung cancer

Firstly, we investigated the associations between the caveolae related genes and the prognosis of LUAD and LUSC patients. We used KM plotter analysis to generate survival curve. The first progression survival (FP), overall survival (OS), post-progression survival (PPS) were summarized in Figure S1, Table S4 and Table S5. It is surprising to find out that the patients with the high expression of CAV1, CAV2, CAV3, CAVIN1 and CAVIN2 tightly correlated with a better prognosis in patients with LUAD (P<0.05), while there were no statistically significances in LUSC patients. Therefore, we focused on the effects of CAVs and CAVINs in patients with LUAD.

**Figure 5:**
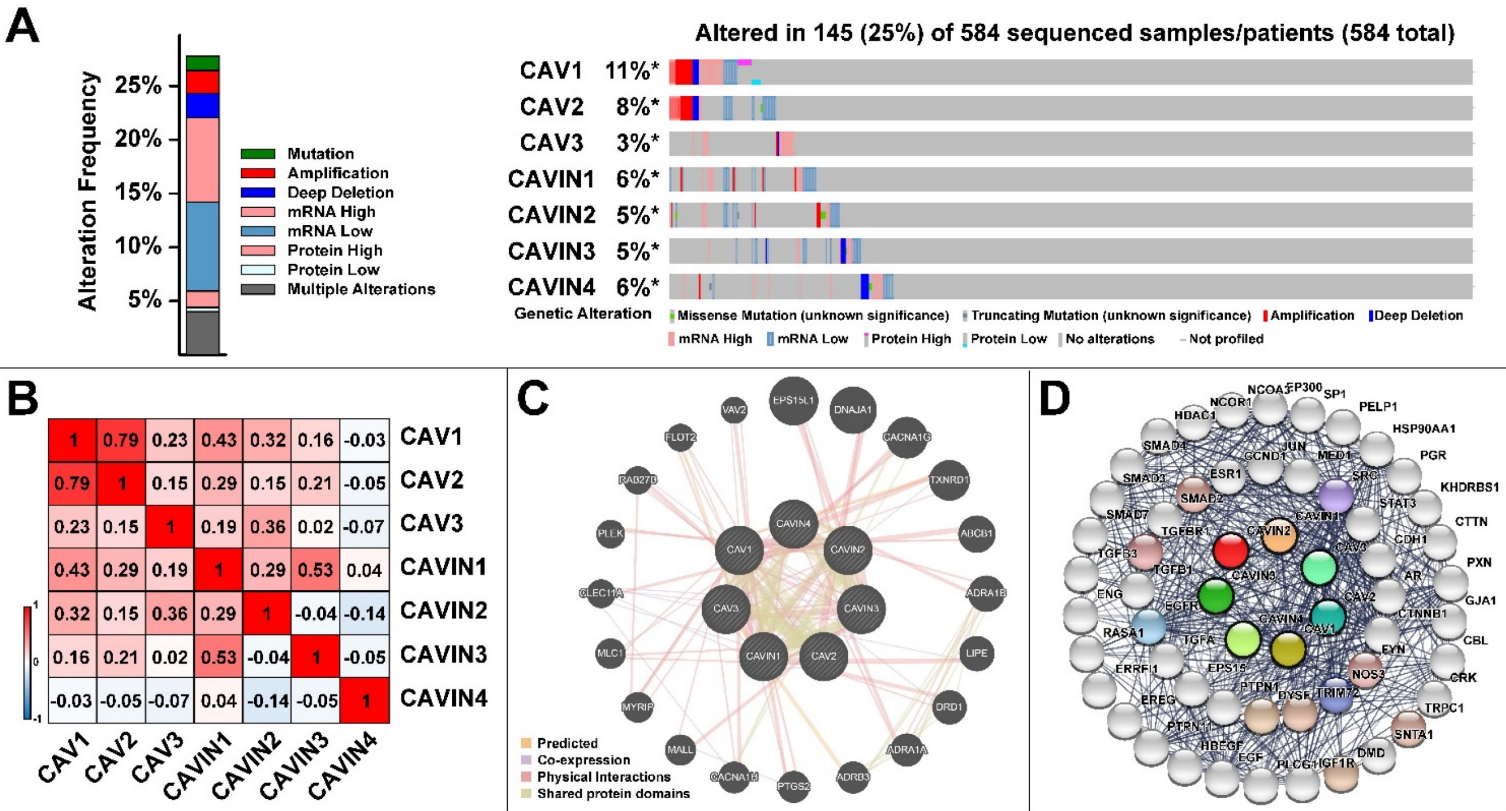
** Molecular characteristics of CAVs and CAVINs in LUAD patients.** A. Gene mutation analysis of CAVs and CAVINs in patients with LUAD by cBioPortal analysis. B. Pearson's correlation analysis of CAVs and CAVINs. C. GeneMANIA analysis of relevant interactive genes of CAVs and CAVINs. D. Molecular network for CAVs and CAVINs and the most frequently altered neighbor genes by STRING.

**Figure 6:**
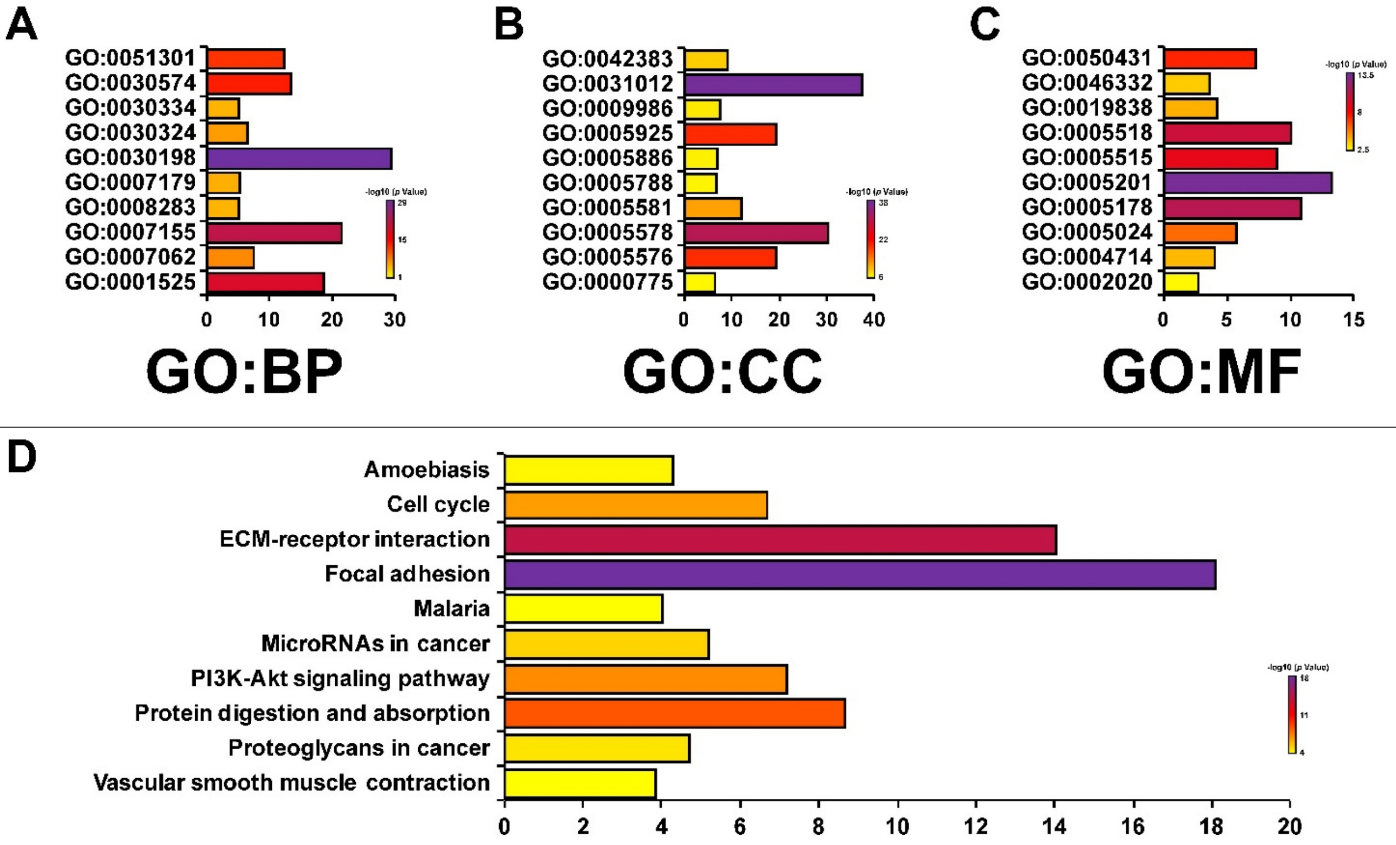
** The enrichment analysis of CAVs and CAVINs with most frequently altered neigh-boring genes.** A, B and C. Gene significantly associated with CAVs and CAVINs alteration by GO enrichment analysis. Biological processes (BP), cellular components (CC) and molecular functions (MF). D. Molecular pathways associated with CAVs and CAVINs using DAVID tools.

Then, in order to analyze the genetic alterations, corrections, networks and related molecular pathways among CAVs and CAVINs, we performed cBioPortal, Gene-MANIA, STRING and DAVID analysis. Figure 5A represented that the genetic characteristics of CAV1 was prominently altered (11%), which was mostly due to the low expression and amplification of mRNA. Moreover, the correlations of CAVs and CAVINs were analyzed by Pearson's correlation, which showed the significant positive corrections between CAV1 and CAV2, CAVIN1 and CAVIN3 (Figure 5B). Further, the interaction network of CAVs and CAVINs and other most frequently changed neighbor genes were performed. It is suggested that CAVs and CAVINs were co-interacted and closely associated with genes involved in focal adhesion and extracellular matrix organization, such as SRC and STAT3 (Figure 5C and 5D). These results were also verified by DAVID database and presented as GO enrichment (Figure 6A-C) and related molecular pathways (Figure 6D). Our results showed that GO: 0007155 (cell adhesion) and GO: 0030198 (extracellular matrix organization) were significantly regulated by caveolae-related genes alterations (Figure 6A). GO: 0031012 (extracellular matrix) and GO: 0005201 (extracellular matrix structural constituent) were significantly influenced by alterations of CAVs and CAVINs (Figure 6B-C). These results indicated that CAVs and CAVINs were involved in the cell homeostasis through extracellular matrix.

### CAVIN2/SDPR inhibit LUAD cells proliferation and migration in vitro

According to the above results, we found that the caveolae-related genes were strongly associated with the prognosis of LUAD patients. We further cross-analyzed the OS of LUAD patients based on CAVs and CAVINs expression levels for intra-family study, respectively. Intra-family comparison showed no matter how much other CAVINs expressed, CAVIN2/SDPR expression levels can distinguish OS of patients with LUAD independent other CAVINs expression levels (Figure S2). Taken together, these results suggest that CAVIN2/SDPR can be used as a prognostic predictor in patients with LUAD.

**Figure 7:**
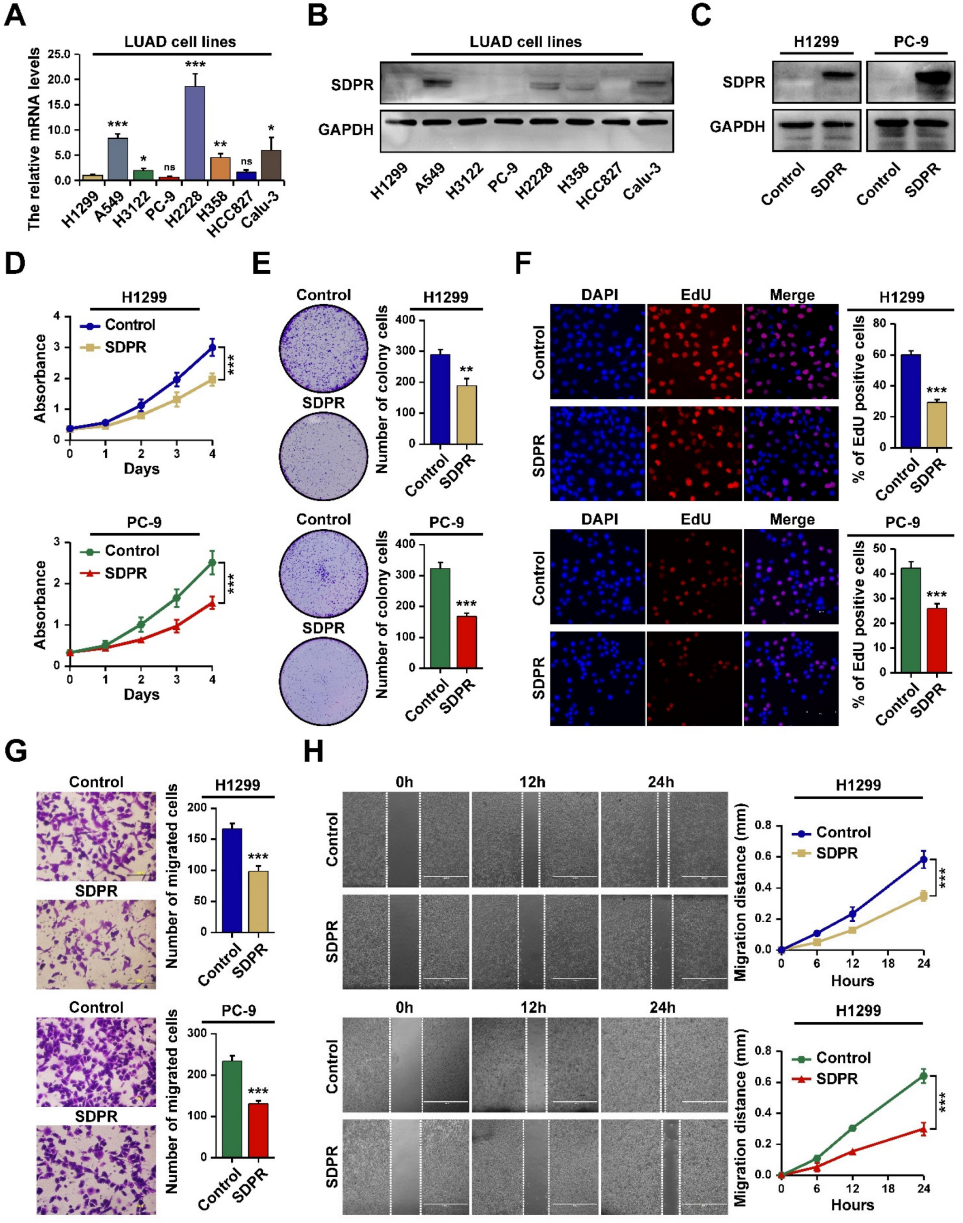
** SDPR inhibits LUAD cells proliferation and migration in vitro.** A. The mRNA expression levels of SDPR in LUAD cell lines determined by RT-qPCR. B. The protein expression levels of SDPR in LUAD cell lines determined by western blot. C. The expression of SDPR in SDPR ex-pressing plasmid transfected H1299 and PC-9 cells determined by western blot. D-F. MTT (D), Colony formation (E) and EdU analysis (F) in SDPR-overexpressed H1299 and PC-9 cells. G-H. Transwell (G) and Scratch analysis (H) of SDPR-overexpressed H1299 and PC-9 cells, as well as control cells. *P <0.05, **P <0.01, ***P < 0.001.

We next performed in vitro experiments to assess the effect of CAVIN2/SDPR (referred to as SDPR) on the biological behaviors in LUAD cells. We firstly determined the expression levels of SDPR in different LUAD cell lines by RT-PCR (Figure 7A) and western blot (Figure 7B). The results showed that SDPR expression was lower in LUAD cell lines, especially in H1299 and PC-9. Next, we assessed whether SDPR overexpression in LUAD cells can influence lung cancer proliferation and migration by SDPR-transfected H1299 and PC-9 cells (Figure 7C). The MTT and colony formation assays indicated that overexpression of SDPR inhibited LUAD cell proliferation (Figure 7D and 7E). The EdU analysis also showed that the number of EdU-positive cells was significantly less in SDPR-overexpressed H1299 and PC-9 cells compared to the control cells (Figure 7F). The transwell and scratch assays indicated that overexpression of SDPR inhibited LUAD cell migration (Figure 7G and 7H). Together, these results indicate that SDPR functioned as a tumor suppressor in LUAD.

### SDPR induce LUAD cells apoptosis and block cells at S phase

**Figure 8:**
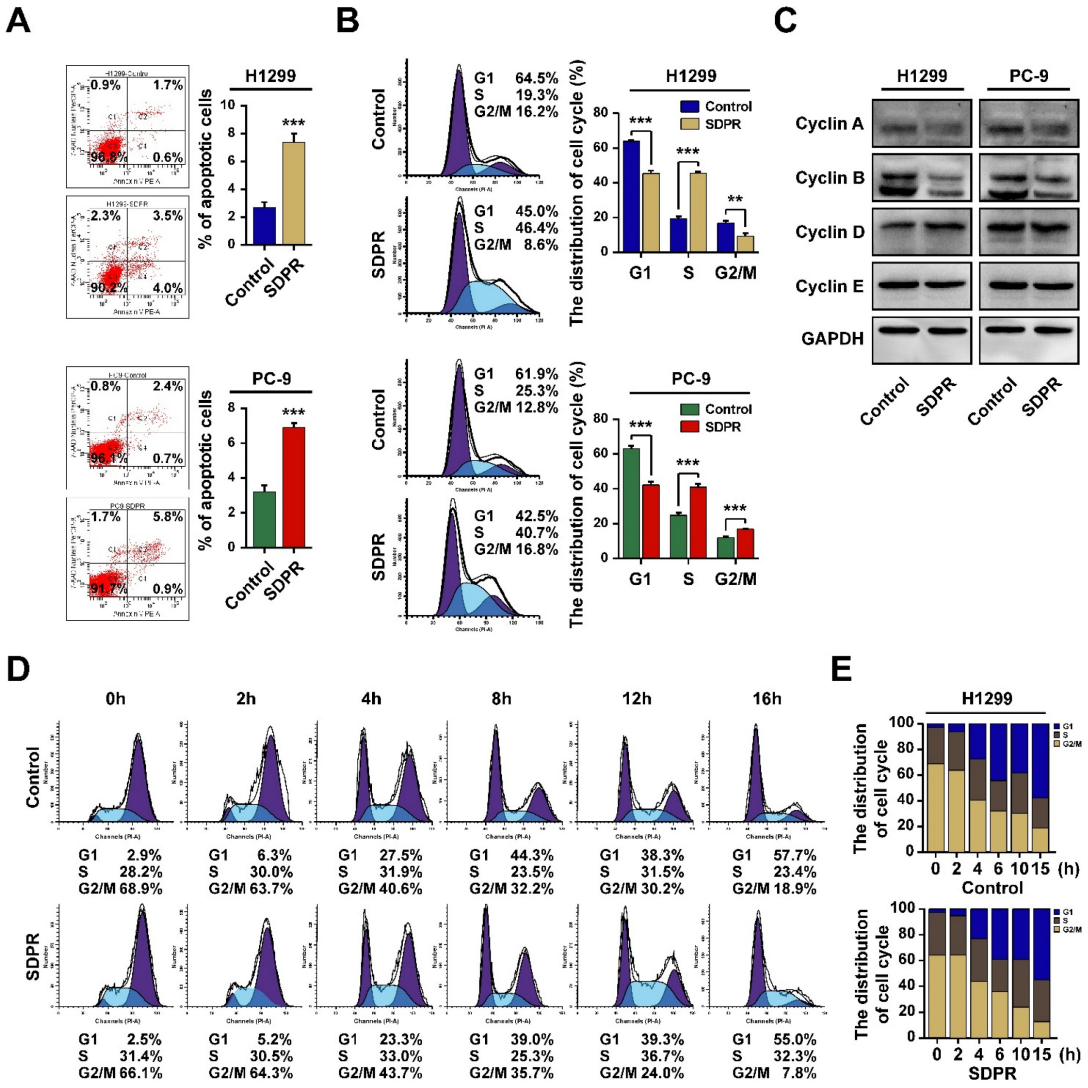
** SDPR induce cell apoptosis and cell cycle arrest at S phase.** A. The cell apoptosis analysis of SDPR-overexpressed H1299, PC-9 cells and control cells determined by flow cytometry analysis. B. The cell cycle distribution of SDPR-overexpressed H1299, PC-9 cells and control cells deter-mined by flow cytometry analysis. C. The expression of cell cycle related proteins determined by western blot. D-E. The cell cycle synchronization analysis of SDPR-overexpressed PC-9 and control cells determined by flow cytometry analysis. **P <0.01, ***P < 0.001.

The cell cycle dysregulation is one of the most frequent alterations associated with the tumor progression. We further observed whether SDPR inhibited LUAD cell proliferation by regulating cell apoptosis or cell cycle. We used flow cytometry analysis to identify the changes after over-expression of SDPR in LUAD cells. The results showed that the number of apoptotic cells was significantly higher in SDPR-overexpressed H1299 and PC-9 cells compared with the control cells (Figure 8A). Furthermore, Flow cytometry analysis revealed SDPR inhibited cell proliferation by arresting cell cycle in S phase and at the same time, the population of cells in the G1 phase was decreased (Figure 8B). In order to reveal the mechanism of SDPR involved in the process of S phase arrest, we then assessed the expression of genes related to cell cycle S phase. The results showed the expression of Cyclin A and Cyclin B was reduced, whereas the expression of Cyclin D and Cyclin E remained unchanged in SDPR-overexpressed H1299 and PC-9 cells (Figure 8C). Further, cells were treated with nocodazole to induce G2/M phase synchronization, (Figure 8D). Results showed that at 16 h after nocodazole treatment, the G2/M percentage for control cells at (18.9 %) was greater than the G2/M percentage for SDPR-overexpressed PC-9 cells (7.8 %). However, the percentage of S stage for control cells (23.4 %) was less than that of SDPR-overexpressed cells (32.3 %). Collectively, these results indicated that SDPR induced cell apoptosis and blocked cells at S phase in LUAD cells.

## Discussion

Accumulating evidence suggested that it is necessary to identify potential biomarkers and targets for the precision therapy in cancer 32, 33. Caveolae-related proteins include coat proteins (caveolins) and adaptor proteins (cavins), as the main component of the fossa, play an important role in a variety of physiological and pathological processes, such as cell endocytosis, maintenance of lipid homeostasis, signal transduction and tumor occurrence and development 34. In past decades, some individual CAVs and CAVINs has been found to play pivotal roles in tumorigenesis 35, 36. Our study comprehensively investigates the expressions and prognosis of all caveolae-related proteins in patients with lung cancer. These findings are expected to provide solid evidence for target therapy towards lung cancer in the future.

According to the multiple large open databases, caveolae-related proteins all exhibited downregulation in lung cancer tissues, which indicated that CAVs and CAVINs may act as tumor suppressors in lung cancer development. However, we found the LUAD patients with the high expression of CAV1, CAV2, CAV3, CAVIN1 and CAVIN2/SDPR tightly correlated with a better prognosis, while there were no statistical significances in LUSC. These differences will need more investigations focusing on histologically specific tumorigenesis in the future.

Further genetic analysis indicated frequent genetic alterations in the CAVs and CAVINs that are differentially expressed in LUAD. We also found that the locations of CAVs and CAVINs were closely relevant to their functions, as the altered levels of these genes mainly influence molecules and pathways involved in focal adhesion, ECM-receptor interaction and TGF-β related genes. Future studies can focus on these signaling pathways to uncover the association of caveolae-related genes to the biological properties of cancer cells.

CAVIN2/SDPR, as a member of cavin family, was firstly identified as a phosphatidylserine-binding protein. CAVIN2/SDPR has been studied on the role of regulating caveolae formation and inducing membrane curvature 37. It has been reported that CAVIN2/SDPR is a potential diagnostic indicator in cancers such as hepatocellular carcinoma and gastric cancer 38, 39. In the current study, we further found that CAVIN2/SDPR can be used as a prognostic predictor independent of other CAVINs in patients with LUAD. Furthermore, we demonstrated that CAVIN2/SDPR can function as a tumor suppressor in LUAD. Dysregulation of CAVIN2/SDPR has been reported to play vital roles in a variety of human cancers 38. Consistent with previous study, we demonstrated that CAVIN2/SDPR inhibited LUAD proliferation and migration. Moreover, overexpression of CAVIN2/SDPR induced cell cycle arrest at S phase, resulting in the inhibition of proliferation and induction of apoptosis.

## Conclusions

In conclusion, we systematically analyzed the differential expression and prognostic value of caveolae-related genes in lung cancer. Our study revealed that SDPR functioned as a tumor suppressor, and may serve as prognostic biomarkers in patients with LUAD. Mechanically, the overexpression of SDPR inhibited cell proliferation by inducing cell apoptosis and blocking cells at S phase in LUAD cells. Collectively, our findings provided new insights into the molecular pathogenesis correlation between SDPR and LUAD and may constitute a promising novel therapeutic target for blocking progression of LUAD.

## Supplementary Material

Supplementary figures and tables.
