# Supplementary material for: CAVIN2/SDPR Functioned as a Tumor Suppressor in Lung Adenocarcinoma from Systematic Analysis of Caveolae-Related Genes and Experimental Validation
Source: J Cancer. 2023 Jul 3;14(11):2001–14. doi: 10.7150/jca.84567 (PMC10367915; doi:10.7150/jca.84567)
Supplement: Supplementary file 1 — Supplementary figures and tables. [file jcav14p2001s1.pdf]

1 Supplementary Materials

Gene		Primers
CAV1	Forward	5'- GAAAGAAGATGGGGGAGGAG -3'
	Reverse	5'- AAAGTCCCCAAAGGCAGAAT -3'
CAV2	Forward	5'- ACGACTCCTACAGCCACCAC-3'
	Reverse	5'- CGTCCTACGCTCGTACACAA -3'
CAVIN1	Forward	5'- GCTCCTTCCGAACTTCCTCT -3'
	Reverse	5'- ACTTGGACAACCAGGACAGG -3'
CAVIN2	Forward	5'- CTTGTGCCTTGTCCCAAAAT-3'
	Reverse	5'- CGCGTAGCTACCCTCATAGC-3'
CAVIN3	Forward	5'- CTTGTGCCTTGTCCCAAAAT-3'
	Reverse	5'- TTATTGATGGTGAGCGCAAG-3'
GAPDH	Forward	5'-GTCTCCTCTGACTTCAACAGCG-3'
	Reverse	5'-ACCACCCTGTTGCTGTAGCCAA-3'

Table S1. RT-PCR primers of mRNA.

	Type of Lung Cancer versus Normal Lung Tissue	Fold Change	p Value	t Test	Source and/or Reference
CAV1	Lung Adenocarcinoma	-7.455	9.31E-15	-13.64	Garber Lung Statistics[40]
	Squamous Cell Lung Carcinoma	-8.628	2.26E-10	-12.655	Garber Lung Statistics[40]
	Lung Adenocarcinoma	-4.377	2.69E-33	-22.083	Okayama Lung Statistics[41]
	Lung Adenocarcinoma	-11.725	2.59E-46	-26.261	Selamat Lung Statistics[42]
	Lung Adenocarcinoma	-8.702	3.48E-17	-15.689	Beer Lung Statistics[43]
	Lung Adenocarcinoma	-6.393	2.17E-33	-19.214	Landi Lung Statistics[44]
	Lung Adenocarcinoma	-18.95	7.63E-10	-9.996	Bhattacharjee Lung Statistics[45]
	Lung Carcinoid Tumor	-66.005	2.10E-13	-13.285	Bhattacharjee Lung Statistics[45]
	Squamous Cell Lung Carcinoma	-6.689	8.96E-06	-4.954	Bhattacharjee Lung Statistics[45]
	Small Cell Lung Carcinoma	-41.875	3.19E-05	-7.043	Bhattacharjee Lung Statistics[45]
	Squamous Cell Lung Carcinoma	-7.84	9.87E-06	-11.034	Wachi Lung Statistics[46]
	Lung Adenocarcinoma	-7.611	1.70E-12	-11.077	Su Lung Statistics[47]
	Lung Adenocarcinoma	-6.292	7.94E-08	-7.838	Stearman Lung Statistics[48]
	Lung Adenocarcinoma	-5.163	2.38E-16	-11.605	Hou Lung Statistics[49]
	Large Cell Lung Carcinoma	-12.444	3.87E-11	-12.59	Hou Lung Statistics[49]
	Squamous Cell Lung Carcinoma	-5.36	1.09E-14	-13.091	Hou Lung Statistics[49]
CAV2	Lung Adenocarcinoma	-5.827	3.83E-17	-13.553	Su Lung Statistics[47]
	Lung Adenocarcinoma	-7.136	7.87E-40	-20.238	Selamat Lung Statistics[42]
	Lung Adenocarcinoma	-4.042	5.54E-25	-19.166	Okayama Lung Statistics[41]
	Small Cell Lung Carcinoma	-13.845	4.18E-05	-9.871	Garber Lung Statistics[40]
	Lung Adenocarcinoma	-3.67	3.06E-07	-8.587	Garber Lung Statistics[40]
	Squamous Cell Lung Carcinoma	-4.533	2.62E-06	-7.611	Garber Lung Statistics[40]
	Lung Adenocarcinoma	-5.513	3.22E-29	-16.102	Landi Lung Statistics[44]
	Small Cell Lung Carcinoma	-23.658	3.24E-06	-7.141	Bhattacharjee Lung Statistics[45]
	Lung Carcinoid Tumor	-36.247	1.36E-10	-11.498	Bhattacharjee Lung Statistics[45]
	Lung Adenocarcinoma	-7.355	1.39E-06	-6.345	Bhattacharjee Lung Statistics[45]

	Squamous Cell Lung Carcinoma	-7.418	8.83E-05	-8.186	Wachi Lung Statistics[46]
	Lung Adenocarcinoma	-4.484	2.13E-07	-7.29	Stearman Lung Statistics[48]
	Lung Adenocarcinoma	-5.732	1.17E-16	-11.184	Hou Lung Statistics[49]
	Large Cell Lung Carcinoma	-19.139	6.98E-13	-14.389	Hou Lung Statistics[49]
	Squamous Cell Lung Carcinoma	-4.971	9.02E-17	-12.858	Hou Lung Statistics[49]
CAV3	Squamous Cell Lung Carcinoma	-2.51	1.11E-07	-6.711	Talbot Lung Statistics[50]
	Lung Adenocarcinoma	-3.419	7.56E-14	-10.765	Okayama Lung Statistics[41]
Cavin-1/PTRF	Lung Adenocarcinoma	-3.058	3.84E-14	-14.102	Stearman Lung Statistics[48]
	Small Cell Lung Carcinoma	-7.237	2.24E-06	-6.124	Bhattacharjee Lung Statistics[45]
	Lung Adenocarcinoma	-6.346	2.87E-06	-6.301	Bhattacharjee Lung Statistics[45]
	Lung Carcinoid Tumor	-23.573	2.11E-09	-8.083	Bhattacharjee Lung Statistics[45]
	Squamous Cell Lung Carcinoma	-3.706	0.001	-3.194	Bhattacharjee Lung Statistics[45]
	Lung Adenocarcinoma	-2.317	9.71E-07	-7.041	Garber Lung Statistics[40]
	Squamous Cell Lung Carcinoma	-2.652	1.17E-05	-5.897	Garber Lung Statistics[40]
	Lung Adenocarcinoma	-2.625	1.54E-11	-9.091	Su Lung Statistics[47]
	Lung Adenocarcinoma	-2.453	4.10E-25	-13.567	Landi Lung Statistics[44]
	Squamous Cell Lung Carcinoma	-2.385	2.67E-04	-6.104	Wachi Lung Statistics[46]
	Lung Adenocarcinoma	-3.176	1.84E-23	-13.813	Selamat Lung Statistics[42]
	Lung Adenocarcinoma	-2.46	2.38E-13	-9.084	Hou Lung Statistics[49]
	Large Cell Lung Carcinoma	-4.291	5.89E-10	-10.17	Hou Lung Statistics[49]
	Squamous Cell Lung Carcinoma	-2.172	1.17E-11	-8.974	Hou Lung Statistics[49]
Cavin-2/SDPR	Large Cell Lung Carcinoma	-9.981	2.64E-05	-8.583	Garber Lung Statistics[40]
	Squamous Cell Lung Carcinoma	-11.994	6.95E-08	-10.07	Garber Lung Statistics[40]
	Lung Adenocarcinoma	-9.404	8.81E-07	-10.589	Garber Lung Statistics[40]
	Small Cell Lung Carcinoma	-15.408	9.39E-04	-6.61	Garber Lung Statistics[40]
	Lung Adenocarcinoma	-5.976	9.18E-24	-18.099	Okayama Lung Statistics[41]
	Lung Adenocarcinoma	-6.617	2.13E-34	-18.484	Selamat Lung Statistics[42]
	Squamous Cell Lung Carcinoma	-5.833	3.81E-05	-8.033	Wachi Lung Statistics[46]
	Lung Adenocarcinoma	-3.803	2.43E-11	-8.227	Su Lung Statistics[47]
	Lung Adenocarcinoma	-4.783	1.02E-22	-13.571	Landi Lung Statistics[44]
	Squamous Cell Lung Carcinoma	-8.845	7.39E-23	-16.666	Hou Lung Statistics[49]
	Lung Adenocarcinoma	-7.204	4.79E-16	-11.068	Hou Lung Statistics[49]
	Large Cell Lung Carcinoma	-10.929	2.30E-12	-12.367	Hou Lung Statistics[49]
Cavin-3/SRBC /PRKCDBP	Lung Adenocarcinoma	-2.337	2.27E-14	-8.996	Selamat Lung Statistics[42]
Cavin-4/MURC	NA	NA	NA	NA	NA

Table S2. The Changes of CAVs and CAVINs Expression in Transcription Level between Different Types of Lung Cancer and Normal Lung Tissues by Oncomine Database.

LUAD						
	CAV1	Age	Gender	STAS	T	Stage
Correlation Coefficient	1.000	-0.282	-0.250	-0.318	**-0.591**	**-0.591**
Sig.（2-Tailed）	.	0.229	0.287	0.172	0.006	0.006
N	20	20	20	20	20	20
	CAV2	Age	Gender	STAS	T	Stage

Correlation Coefficient	1.000	-0.318	-0.050	**-0.491**	**-0.451**	**-0.451**
Sig.（2-Tailed）	.	0.172	0.834	0.028	0.046	0.046
N	20	20	20	20	20	20
	CAVIN1	Age	Gender	STAS	T	Stage
Correlation Coefficient	1.000	**-0.445**	-0.150	-0.087	**-0.551**	**-0.551**
Sig.（2-Tailed）	.	0.049	0.527	0.716	0.012	0.012
N	20	20	20	20	20	20
	CAVIN2	Age	Gender	STAS	T	Stage
Correlation Coefficient	1.000	-0.355	-0.190	-0.116	**-0.751**	**-0.751**
Sig.（2-Tailed）	.	0.125	0.422	0.627	0.000	0.000
N	20	20	20	20	20	20
	CAVIN3	Age	Gender	STAS	T	Stage
Correlation Coefficient	1.000	-0.318	-0.190	-0.058	-0.150	-0.150
Sig.（2-Tailed）	.	0.172	0.422	0.809	0.527	0.527
N	20	20	20	20	20	20
**LUSC**						
	CAV1	Age	Gender	STAS	T	Stage
Correlation Coefficient	1.000	0.390	.	**-0.731**	-0.392	-0.392
Sig.（2-Tailed）	.	0.089	.	0.000	0.087	0.087
N	20	20	20	20	20	20
	CAV2	Age	Gender	STAS	T	Stage
Correlation Coefficient	1.000	0.030	.	-0.37	**-0.706**	**-0.706**
Sig.（2-Tailed）	.	0.900	.	0.108	0.001	0.001
N	20	20	20	20	20	20
	CAVIN1	Age	Gender	STAS	T	Stage
Correlation Coefficient	1.000	0.030	.	-0.230	-0.357	-0.357
Sig.（2-Tailed）		0.900	.	0.329	0.122	0.122
N	20	20	20	20	20	20
	CAVIN2	Age	Gender	STAS	T	Stage
Correlation Coefficient	1.000	-0.150	.	**-0.511**	**-0.566**	**-0.566**
Sig.（2-Tailed）		0.527	.	0.021	0.009	0.009
N	20	20	20	20	20	20
	CAVIN3	Age	Gender	STAS	T	Stage
Correlation Coefficient	1.000	0.330	.	-0.390	-0.288	-0.288
Sig.（2-Tailed）		0.155	.	0.089	0.219	0.219
N	20	20	20	20	20	20

STAS, spread through air spaces; T, tumor size; Sig, significance; N, number.

Table S3. The Correlation analysis between CAVs/CAVINs and clinicopathological factors of patients by spearman method.

Gene	Dataset/ Affymetrix ID	Survival outcome	No.of cases	HR	95% CI	*p*-value
CAV1	212097_at	FP	461	0.43	0.31-0.6	**2.7E-07**
		OS	719	0.6	0.48-0.77	**2.5E-05**

		PPS	125	0.75	0.47-1.19	0.22
CAV2	203323_at	FP	461	0.52	0.38-0.72	**5.6E-05**
		OS	719	0.59	0.46-0.74	**8.2E-06**
		PPS	125	0.74	0.47-1.18	0.21
CAV3	208204_s_at	FP	461	0.65	0.48-0.89	**0.0072**
		OS	719	0.74	0.59-0.94	**0.013**
		PPS	125	1.38	0.86-2.2	0.18
CAVIN1	*208789_at*	FP	461	0.92	0.67-1.25	0.59
		OS	719	1.45	1.15-1.84	**0.0015**
		PPS	125	1.2	0.75-1.91	0.46
CAVIN2	222717_at	FP	443	0.36	0.25-0.5	**8.8E-10**
		OS	672	0.44	0.34-0.57	**6.9E-11**
		PPS	115	0.86	0.53-1.4	0.54
CAVIN3	213010_at	FP	461	1.25	0.92-1.71	0.16
		OS	719	1.24	0.98-1.56	0.071
		PPS	125	1.02	0.64-1.63	0.93
CAVIN4	241749_at	FP	443	1.27	0.92-1.76	0.14
		OS	672	0.86	0.67-1.09	0.22
		PPS	115	0.78	0.48-0.27	0.32

Table S4. The prognostic value of CAVs and CAVINs in patients with LUAD.

Gene	Dataset/ Affymetrix ID	Survival outcome	No.of cases	HR	95% CI	*p*-value
CAV1	212097_at	FP	141	0.81	0.48-1.34	0.41
		OS	524	0.93	0.73-1.17	0.53
		PPS	20	1.29	0.46-3.59	0.63
CAV2	203323_at	FP	141	1.13	0.68-1.88	0.65
		OS	524	0.92	0.73-1.17	0.51
		PPS	20	0.77	0.28-2.14	0.62
CAV3	208204_s_at	FP	141	0.7	0.42-1.17	0.17
		OS	524	1.03	0.81-1.31	0.79
		PPS	20	1.12	0.39-3.24	0.84
CAVIN1	*208789_at*	FP	141	0.82	0.49-1.38	0.46
		OS	524	0.98	0.77-1.24	0.88
		PPS	20	0.85	0.31-2.37	0.76
CAVIN2	222717_at	FP	141	0.81	0.48-1.34	0.41
		OS	271	0.89	0.65-1.22	0.47
		PPS	20	0.9	0.32-2.52	0.85
CAVIN3	213010_at	FP	141	0.85	0.51-1.42	0.53
		OS	524	1.02	0.81-1.3	0.84
		PPS	20	0.69	0.24-1.98	0.48

CAVIN4	241749_at	FP	141	1.47	0.88-2.46	0.14
		OS	271	1.13	0.83-1.54	0.44
		PPS	20	1.02	0.37-2.84	0.97

Table S5. The prognostic value of CAVs and CAVINs in patients with LUSC cancer.

Figure S1

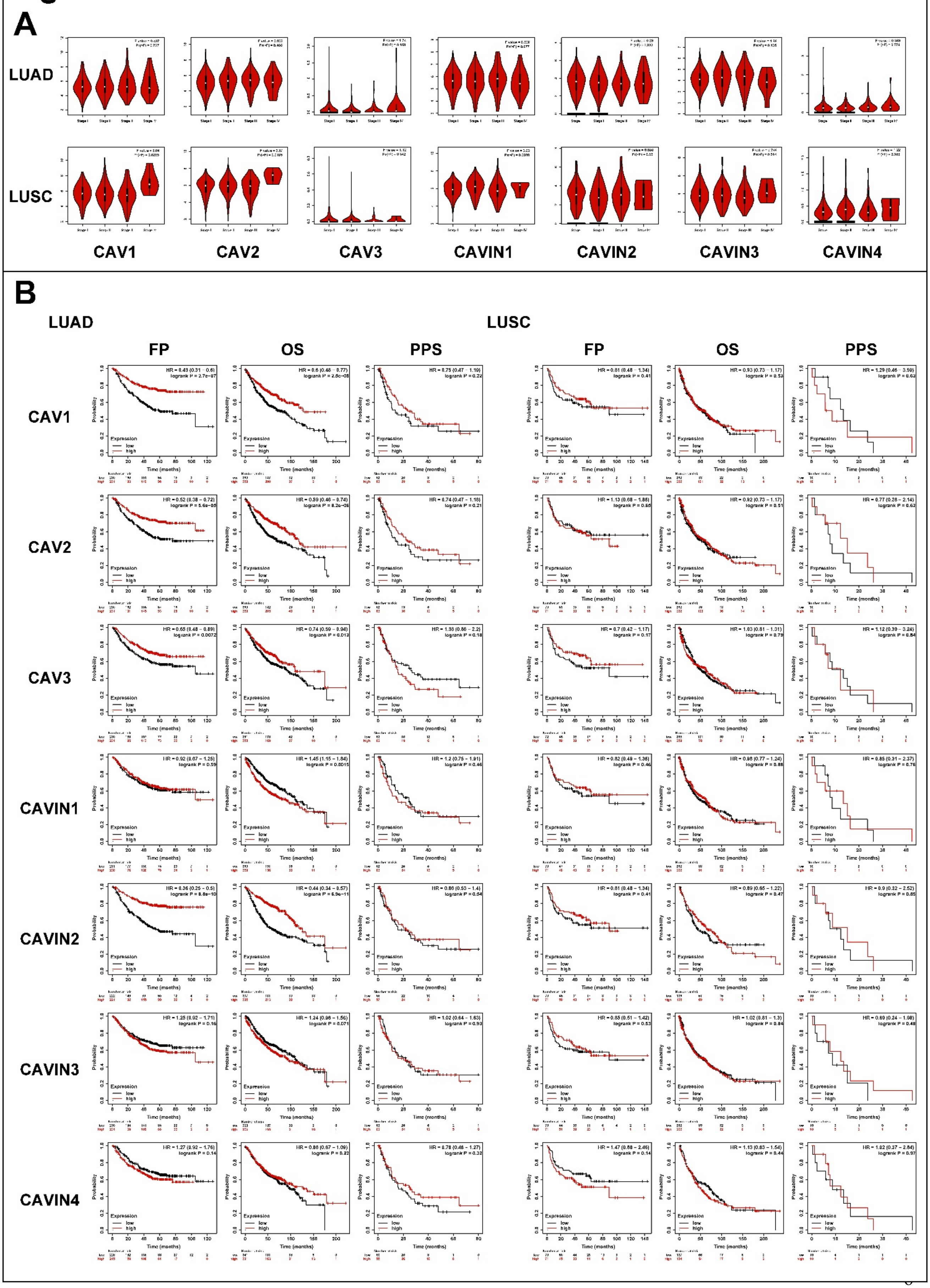
A
LUAD
LUSC
CAV1
CAV2
CAV3
CAVIN1
CAVIN2
CAVIN3
CAVIN4
B
LUAD
LUSC
FP
OS
PPS
CAV1
CAV2
CAV3
CAVIN1
CAVIN2
CAVIN3
CAVIN4

Figure S1. The prognostic value of CAVs and CAVINs in patients with LUAD cancer (A) and LUSC (B).

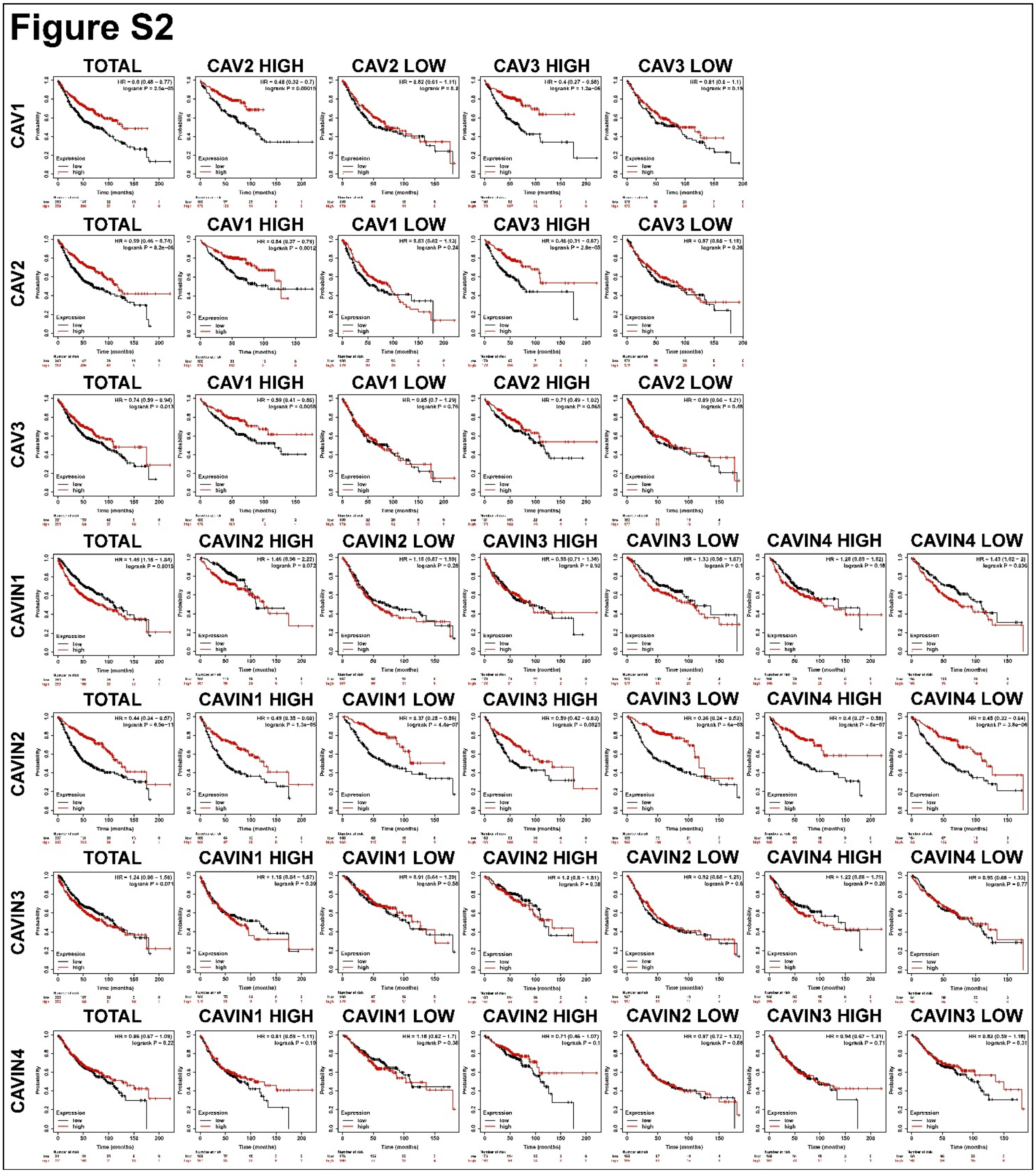

Figure S2. The levels of CAVs and CAVINs on OS of patients in LUAD using intra-family study.

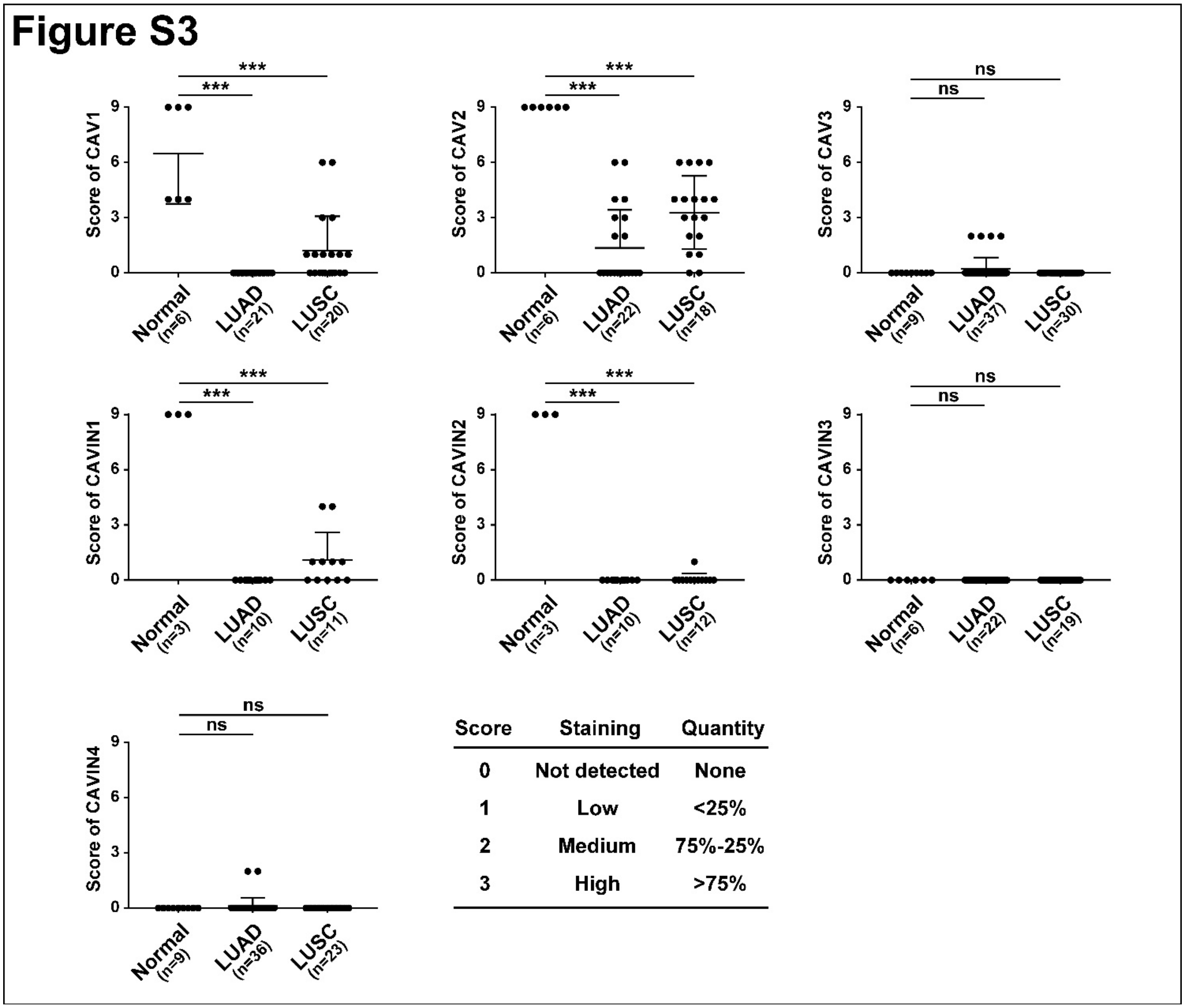

Score	Staining	Quantity
0	Not detected	None
1	Low	<25%
2	Medium	75%-25%
3	High	>75%

Figure S3. The score of expression levels of CAVs and CAVINs from human protein atlas. *P <0.05, **P <0.01, ***P < 0.001.
